# Towards a Cost-Effective Implementation of Genomic Prediction Based on Low Coverage Whole Genome Sequencing in Dezhou Donkey

**DOI:** 10.3389/fgene.2021.728764

**Published:** 2021-11-03

**Authors:** Changheng Zhao, Jun Teng, Xinhao Zhang, Dan Wang, Xinyi Zhang, Shiyin Li, Xin Jiang, Haijing Li, Chao Ning, Qin Zhang

**Affiliations:** ^1^ Shandong Provincial Key Laboratory of Animal Biotechnology and Disease Control and Prevention, College of Animal Science and Veterinary Medicine, Shandong Agricultural University, Tai’an, China; ^2^ National Engineering Research Center for Gelatin-based TCM, Dong-E E-Jiao Co., Ltd., Dong’e County, China

**Keywords:** dezhou donkey, low coverage whole genome sequencing, genotype imputation, genomic prediction, GBLUP

## Abstract

Low-coverage whole genome sequencing is a low-cost genotyping technology. Combined with genotype imputation approaches, it is likely to become a critical component of cost-effective genomic selection programs in agricultural livestock. Here, we used the low-coverage sequence data of 617 Dezhou donkeys to investigate the performance of genotype imputation for low-coverage whole genome sequence data and genomic prediction based on the imputed genotype data. The specific aims were as follows: 1) to measure the accuracy of genotype imputation under different sequencing depths, sample sizes, minor allele frequency (MAF), and imputation pipelines and 2) to assess the accuracy of genomic prediction under different marker densities derived from the imputed sequence data, different strategies for constructing the genomic relationship matrixes, and single-vs. multi-trait models. We found that a high imputation accuracy (>0.95) can be achieved for sequence data with a sequencing depth as low as 1x and the number of sequenced individuals ≥400. For genomic prediction, the best performance was obtained by using a marker density of 410K and a G matrix constructed using expected marker dosages. Multi-trait genomic best linear unbiased prediction (GBLUP) performed better than single-trait GBLUP. Our study demonstrates that low-coverage whole genome sequencing would be a cost-effective approach for genomic prediction in Dezhou donkey.

## Introduction

Dezhou donkey, originating from Dezhou area, Shandong Province, China, is one of the major donkey breeds in China. It is famous for its large body size (and thus good meat production ability) and excellent skin quality (for producing donkey-hide gelatin). It has been introduced as a breeding stock into many areas of China and has also brought considerable economic benefits to farmers (Wang et al., 2020a). Therefore, Dezhou donkey plays an important role in the donkey industry in China. However, selective breeding based on animal breeding theory had never been practiced in Dezhou donkey in the past. In recent years, along with the increased importance of the donkey industry in livestock agriculture in China, selective breeding is gradually becoming an important issue in donkey production, and some breeding work is being carried out in the Dezhou donkey population.

Starting with the pioneered work of Meuwissen et al. (2001), genomic selection (GS) has been widely used in selective breeding in almost all major farm animal species and has brought great increases of genetic progress and economic benefit for many animal breeding industries (Schaeffer, 2006; Stock and Reents, 2013; Wiggans et al., 2017). Typically, GS is carried out using a high-density (or medium-density) single-nucleotide polymorphism (SNP) array. Many commercial SNP arrays have been developed for major farm animal species (Stock and Reents, 2013). However, there are still some species, such as donkey, for which no such array is available, which inhibits the application of GS in these species.

Recently, along with the rapid development of next-generation sequencing technology and reduction of sequencing cost, GS using genotypes revealed by whole genome sequencing (WGS), instead of SNP array, has drawn interests of animal GS community (Hickey 2013; Daetwyler et al., 2014; Georges 2014). The motivations of using whole genome sequence data are to increase the selection accuracy, to facilitate GS across breeds/populations, and to improve persistence of accuracy across generations (Meuwissen and Goddard, 2010; Hayes et al., 2013). To capture the whole genome variants, a sequencing depth of 10x to 20x is generally required (Rashkin et al., 2017; Jiang et al., 2019). However, at present, sequencing with such depth is still too expensive for a large-scale GS application. An alternative is to perform low-coverage whole genome sequencing (lcWGS) at about 1x or less, and then recovering the missing genotypes by imputation to ensure that all individuals have genotypes for a shared set of variants. This approach has been used in human and some animal species for genome-wide association studies and genomic selection/prediction and proved to be a feasible alternative to normal sequencing (Pasaniuc et al., 2012; Nicod et al., 2016; Liu et al., 2018; Zhang et al., 2021). Since the cost of lcWGS can even be lower than that of a SNP array (e.g., in China, the current price for sequencing a cattle genome at 1x is about ¥ 250 RMB per sample, while the price for genotyping with the Neogen GGP Bovine 100k SNP array is ¥ 280 RMB per sample), it is considered as a cost-effective genotyping approach for GS [referred to as GS 2.0 by Hickey (2013)].

A critical issue of lcWGS-based GS is the accuracy of imputation of missing genotypes, which is affected by several factors, such as sequencing depth, sample size, minor allele frequency (MAF), and imputation method. A number of imputation methods for lcWGS data have been proposed (Davies et al., 2016; Ros-Freixedes et al., 2017; Hui et al., 2020). However, most of these methods require a high-density reference haplotype panel, which is not available for most animal species, including donkey. Davies et al. (2016) proposed a method called STITCH for imputation without requiring a reference haplotype panel. It makes use of the fact that SNPs in sequences are not independent of each other, and it constructs founder haplotypes directly from the sequencing read data and then perform imputation based on a hidden Markov model. This method provides an opportunity of using lcWGS technology for species for which a reference haplotype panel is not available.

In this study, we evaluated the imputation accuracy of lcWGS data with respect to different sequencing depths, sample sizes, MAFs, and imputation pipelines using 617 Dezhou donkey animals that were sequenced with an average depth of 3.5x. We then used the imputed genotypes to investigate the performance of genomic selection for birth weight (BW) and weaning weight (WW) in the Dezhou donkey population under different marker densities, strategies for constructing genomic relationship matrices, and single-vs. two-trait models.

## Materials and Methods

### Animals

The animals used in this study were from the Dong-E E-Jiao Donkey Farm in Shandong Province, China. Animals that had records on both BW and WW were selected. These animals along with their known parents formed the study population for this research, which consisted of 617 animals, of which 594 had records on both traits. These 594 animals (303 males and 291 females) were born between 2015 and 2019. The animals were weaned at 6 months after birth, and their weaning weight was measured at the age of 6 ± 1 month. Weaning weight recorded outside this age range was regarded as invalid record. The means and standard deviations of the two traits were 30.507 ± 4.235 kg (ranging from 15.0 to 42.2 kg) and 116.752 ± 18.227 kg (ranging from 63.5 to 165.5 kg), respectively.

Blood samples were collected from all these animals. Total DNA was isolated using the QIAamp DNA Investigator Kit (QIAGEN, Hilden, Germany) and following the manufacturer’s instruction. DNA quality was evaluated by spectrophotometry and agarose gel electrophoresis.

All of the above experiments were carried out according to the guideline of the experimental animal management of Shandong Agricultural University (SDAUA-2018-018).

### Low-Coverage Whole Genome Sequencing

DNA templates were ultrasonically sheared using a Covaris E220 (Covaris, Woburn, MA, United States) to yield to 150-bp fragments and then prepared for sequencing libraries following the workflow of the NEBNext Ultra DNA Library Preparation Protocol. Multiple Ampure Bead XP cleanups (Beckman Coulter, Brea, CA, United States) were conducted to remove any adapter dimer that might have developed. The quality and concentration of libraries were determined on an Agilent Bioanalyzer 2,100 (Agilent Technologies, Santa Clara, CA). The genomic library for each sample was PE150 sequenced using the Illumina NovaSeq 6,000 sequencing system.

Read quality was assessed using the FastQC software (https://www.bioinformatics.babraham.ac.uk/projects/fastqc/) with focus on base quality scores (*q* > 30), GC content (skewness <5%), N content (<5%), and sequence duplication levels (<100). The resulting data reached a nucleotide length of 150 bp and a base quality score of higher than 30 and were aligned to the donkey reference genome (Wang et al., 2020b) by BWA (Li and Durbin, 2009). SAMtools (Li et al., 2009) was used to transfer the formats and sort and index files. The 617 animals had an average sequencing depth of 3.5x (ranging from 1.9x to 6.4x) (Supplementary Figure S1).

### Pipelines for Genotype Imputation

We compared two imputation pipelines, i.e., Bcftools + Beagle and BaseVar + STITCH. In the first pipeline, we called SNPs using Bcftools (Li, 2011) and then conducted genotype imputation using Beagle v4.1 (Browning and Browning, 2016). In the second pipeline, we called SNPs using BaseVar (Liu et al., 2018) and imputed the missing genotypes (with probabilities) using STITCH v1.6.3. The resulted SNP data from both pipelines were filtered with MAF ≥0.01 and a Hardy–Weinberg equilibrium (HWE) *p*-value > 1e-6 using PLINK (Chang et al., 2015).

### Evaluation of Imputation Accuracy

We evaluated the imputation accuracy under different sequencing depths, sample sizes, and MAFs using the sequence data of additional 18 Dezhou donkey animals provided by the Donkey Research Institute, Liaocheng University, Shandong Province, China. The average sequencing depth of the 18 animals was 13.5x (ranging from 11.2x to 16.3x). Chromosomes 1, 19, and 30, which represented the long, short, and medium chromosomes among the donkey chromosomes, respectively, were chosen to evaluate the imputation accuracy. The imputation accuracy was measured with two criteria, i.e., genotypic concordance and genotypic accuracy. Genotypic concordance is defined as the proportion of correctly imputed genotypes (Fridley et al., 2010), and genotypic accuracy is defined as squared Pearson correlation coefficient (*r* ^2^) between expected dosages (posterior expectation of the imputed allele dosages) and typed genotypes (Browning and Browning, 2009). To evaluate the imputation accuracy for different sequencing depths, in addition to the original sequence data with an average depth of 3.5x, we randomly sampled reads from the sequencing read data to generate sequence data with different lower sequencing depths (0.5x, 1x, 1.5x, and 2x) using Picard (https://broadinstitute.github.io/picard/). For the depths of 0.5x, 1x, and 1.5x, three repeated samplings were performed. To test the effect of sample size (number of low coverage sequenced individuals) on imputation accuracy, three different sample sizes (200, 400, and 617) were considered. The samples with sizes of 200 and 400 were randomly sampled from the 617 animals, and three repeated samplings were performed. To test the effect of MAF on imputation accuracy, we restored the SNPs that were previously filtered out with MAF >0.01 and divided the SNPs into 15 MAF bins: (0–0.001), (0.001–0.002), (0.002–0.005), (0.005–0.01), (0.01–0.02), (0.02–0.05), (0.05–0.1), (0.1–0.15), (0.15–0.2), (0.2–0.25), (0.25–0.3), (0.3–0.35), (0.35–0.4), (0.4–0.45), and (0.45–0.5). The average imputation accuracy in each bin was calculated.

### Genomic Prediction

The imputation-based sequence data was used to investigate the performance of genomic prediction using the 594 animals having records on both BW and WW. The genomic estimated breeding values (GEBVs) were obtained by using the genomic best linear unbiased prediction (GBLUP) method (VanRaden, 2008) under single-trait model as well as two-trait model.

The single-trait GBLUP model is as follows:

y=Xb+Zu+e

where **y** is the vector of observed phenotypes of BW or WW; **b** is the vector of fixed effects, which include the effects of sex, year-seasons when the trait was measured (years for BW: 2015–2019, years for WW: 2016–2019, and four seasons each year), and age (in days, as covariate, for WW) when the trait was measured; **u** is the vector of genomic breeding values with distribution of *N* (0, **G** $\sigma_{a}^{2}$ ), where $\sigma_{a}^{2}$ is the additive genetic variance and **G** is the genomic relationship matrix; **X** and **Z** are the incidence matrices for **b** and **u**, respectively; and **e** is the vector of random residuals with distribution of *N* (0, **I** $\sigma_{e}^{2}$ ).

The two-trait GBLUP model is as follows:

$$\left[\begin{array}{c} y_{1}\\ y_{2} \end{array}\right]=\left[\begin{array}{cc} X_{1} & 0\\ 0 & X_{2} \end{array}\right]\left[\begin{array}{c} b_{1}\\ b_{2} \end{array}\right]+\left[\begin{array}{cc} Z_{1} & 0\\ 0 & Z_{2} \end{array}\right]\left[\begin{array}{c} u_{1}\\ u_{2} \end{array}\right]+\left[\begin{array}{c} e_{1}\\ e_{2} \end{array}\right]$$

where the meanings of the vectors and matrices are the same as those in the single-trait model with the subscripts 1 and 2 referring BW and WW, respectively. It was assumed that $\left[\begin{array}{c} u_{1}\\ u_{2} \end{array}\right]∼N\left(0,G⊗M\right)$ , where **M =** $\left[\begin{array}{cc} \sigma_{a_{1}}^{2} & \sigma_{a_{\mathrm{12}}}\\ \sigma_{a_{\mathrm{12}}} & \sigma_{a_{2}}^{2} \end{array}\right]$ is the variance–covariance matrix of the genomic breeding values of the two traits, and $\left[\begin{array}{c} e_{1}\\ e_{2} \end{array}\right]∼N\left(0,I⊗R\right)$ , where $R=\left[\begin{array}{cc} \sigma_{e_{1}}^{2} & \sigma_{e_{12}}\\ \sigma_{e_{12}} & \sigma_{e_{2}}^{2} \end{array}\right]$ is the residual variance–covariance matrix of the two traits.

Since STITCH provides for each SNP and each individual the imputed genotype (the most likely genotype) as well as the expected genotype dosages (posterior expectation of the genotype dosages), the **G** matrix can be constructed using either the imputed genotypes or the expected genotype dosages. The genotype-based **G** matrix [denoted as **G**(g)] was constructed using the method of VanRaden (2008) as follows:

$$G\left(\text{g}\right)=WW^{\prime }\sum 2p_{j}\left(1-p_{j}\right)$$

where, W is the centralized maker genotype matrix with its ij th element equal to

$$w_{ij}=m_{ij}-2p_{j}$$

where $m_{ij}$ (= 2, 1, or 0) is the original genotype of individual *i* for SNP *j*, and $p_{j}$ is the minor allele frequency of SNP *j*.

For constructing **G** using expected dosages [denoted as **G**(d)], following the idea of the formula for **G**(g), we proposed the following formula:

$$G\left(\text{d}\right)=DD^{\text{'}}/s_{d}$$

where, D is the centralized marker dosage matrix whose elements are zero-centered expected dosages. $s_{d}$ is the sum of variances for every column of D .

To evaluate the effect of marker density on the performance of genomic prediction, we used four levels of marker densities to construct the **G** matrices. From the original sequence data with an average depth of 3.5x, we obtained 2.3M SNPs after imputation and quality control. We then reduced the marker density by down-sampling SNPs from the 2.3M SNPs. We applied linkage disequilibrium (LD) pruning with three LD levels: *r* ^2^ = 0.2, 0.4, and 0.8, by PLINK (Chang et al., 2015), which produced 130, 220, and 410K SNPs, respectively.

In addition, we also evaluated the performance of genomic prediction using the 1x sequencing data, which was sampled from the original sequence data and contained 1.4M SNPs after imputation and quality control.

We used GMAT (Wang et al., 2020a) to construct the **G** matrix. The variance and covariance components involved in the models and GEBVs were estimated by AI-REML using the DMU package (Madsen et al., 2014; http://dmu.agrsci.dk).

### Cross-Validation

In this study, a 12-fold cross-validation (CV) was applied to assess the accuracy of the genomic prediction. The 594 animals were divided into 12 subsets. One of them was taken in turn to be used as a validation population, and the remaining 11 subsets used as a training population. For the two-trait model analysis, we left out the observations on both BW and WW for the animals in the validation set and calculated their GEBVs for both traits simultaneously. The accuracy of genomic prediction for the validation animals was assessed by $r_{{\text{y}}_{\text{c}},\text{GEBV}}$ , the correlation between corrected phenotypic values (*y*_c_) and GEBVs. The corrected phenotype for each animal was calculated as the original phenotypic value corrected for fixed effects [sex, year-season, and age (for WW)], which were estimated by conventional BLUP using the DMU package (Madsen et al., 2014; http://dmu.agrsci.dk). The model for conventional BLUP was the same as that for GBLUP except that the **G** matrix was replaced by the pedigree-based **A** matrix. The bias of predictions was assessed by the regression of *y*_c_ on GEBV ( $b_{{\text{y}}_{\text{c}},\text{GEBV}}$ ), with $b_{{\text{y}}_{\text{c}},\text{GEBV}}=1$ indicating unbiased prediction (Su et al., 2012).

## Results

### Accuracies of Genotype Imputation

#### Comparison of Different Pipelines

**FIGURE 1:**
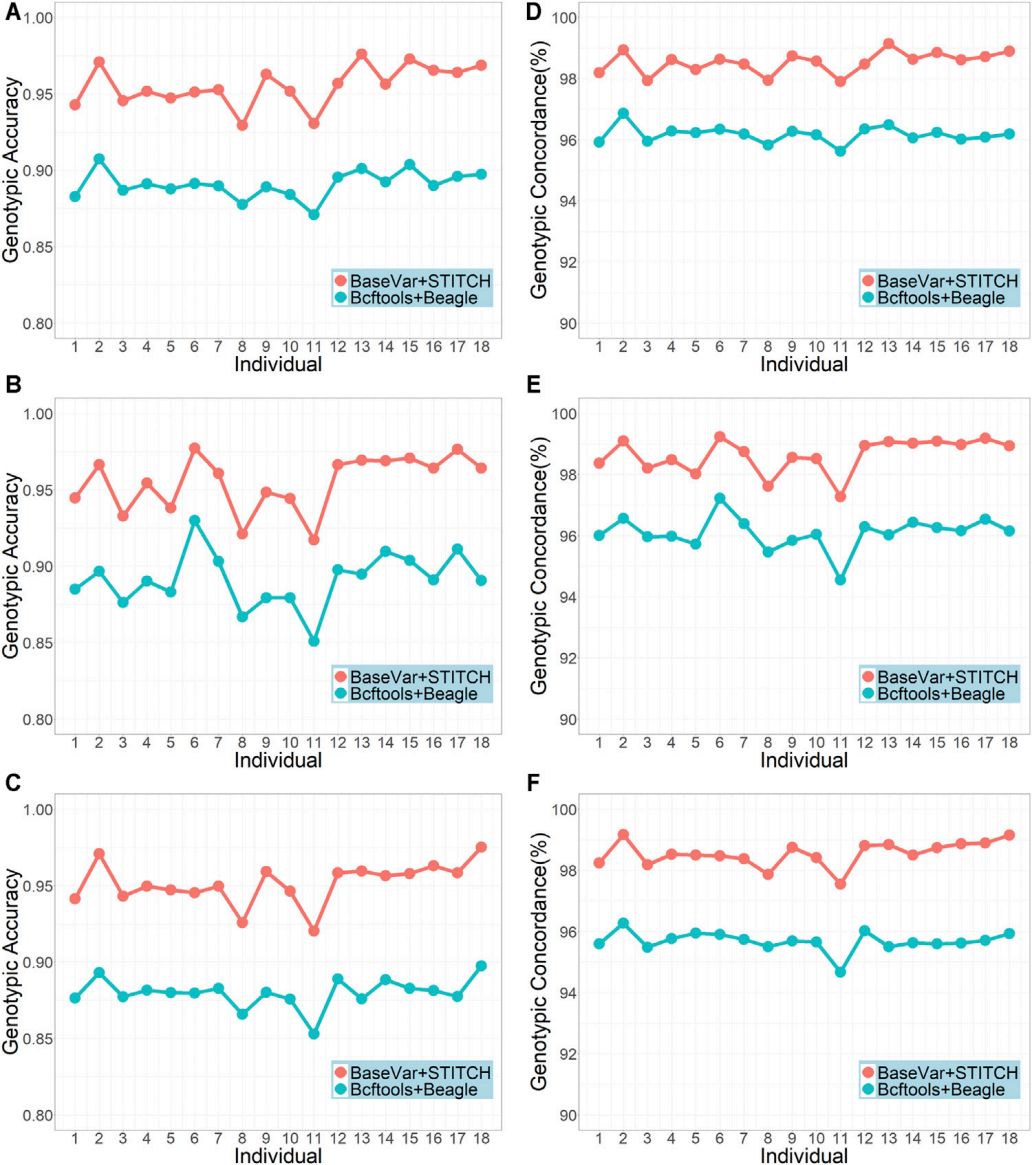
Genotypic accuracy and genotypic concordance using the two imputation pipelines (sample size = 617 and average sequencing depth = 3.5x). **(A–C)** represent genotype accuracy for chromosomes 1, 19, and 30, respectively; **(D–F)** represent genotype concordance for chromosomes 1, 19 and 30, respectively.

The two genotype imputation pipelines, BaseVar + STITCH and Bcftools + Beagle, were compared using the original sequencing data of the 617 animals with an average sequencing depth of 3.5x. Figure 1 shows that the BaseVar + STITCH pipeline was remarkably better than the Bcftools + Beagle pipeline. The average genotypic accuracy from BaseVar + STITCH was about 0.06 higher than that from Bcftools + Beagle, and the average genotypic concordance was about 0.02 higher. Therefore, the BaseVar + STITCH pipeline was used for the subsequent analyses.

#### The Effects of Sample Size and Sequencing Depth

**FIGURE 2:**
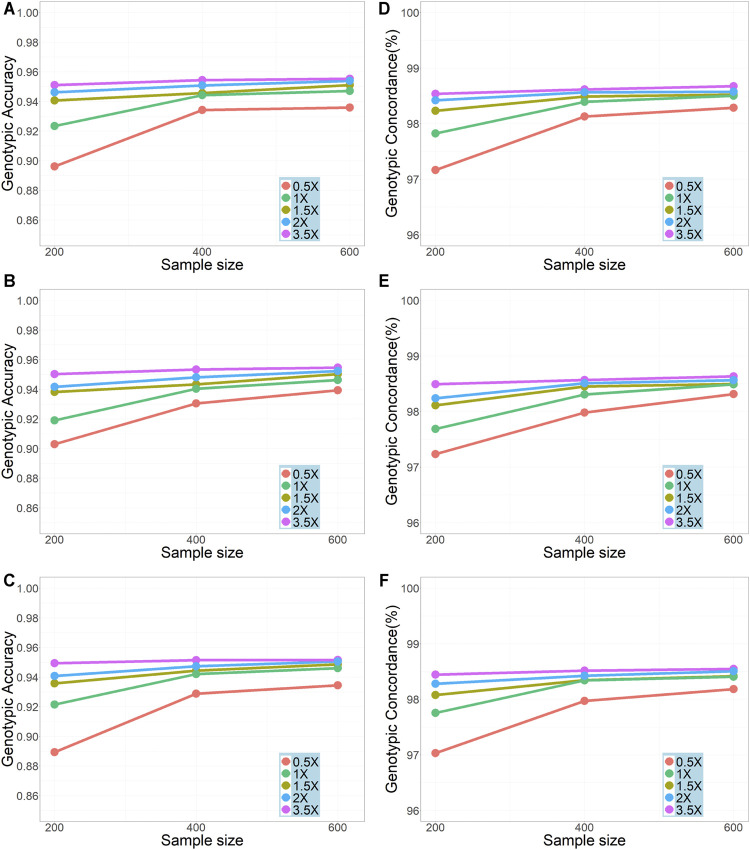
Effects of sample size and sequencing depth on imputation genotypic accuracy and genotypic concordance using the pipeline of BaseVar + STITCH. **(A–C)** represent genotype accuracy for chromosomes 1, 19, and 30, respectively; **(D–F)** represent genotype concordance for chromosomes 1, 19, and 30, respectively.

We compared the genotypic accuracy and genotypic concordance for imputation with different sample sizes (200, 400, and 600) and sequencing depths (0.5x, 1x, 1.5x, 2x, and 3.5x) (Figure 2). In all scenarios, the genotype accuracies were over 0.90 (with only one exception on chromosome 30 in the scenario of sequencing depth = 0.5x and sample size = 200) and the genotypic concordances were over 0.97. In general, as expected, the genotypic accuracy and genotypic concordance increased with the increase of sample size and sequencing depth. The improvement of imputation accuracy was most obvious when the sample size was increased from 200 to 400 and the sequencing depth increased from 0.5x to 1x. For sequencing depths of 0.5x, 1x, and 1.5x, the results from the three repeatedly sampled data were almost the same (see Supplementary Table S1 for chromosome 19 and sample size of 200), so did the results from the repeated samples of sizes 200 and 400 (see Supplementary Table S2). It should be noted that, with a sample size of ≥400, a genotypic accuracy greater than 0.94 and a genotypic concordance greater than 0.98 could be achieved even when the sequencing depth was as low as 1x. However, with a sequencing depth of 0.5x, even for a sample size of 600, the genotype accuracy was less than 0.94.

#### The Effect of MAF

**FIGURE 3:**
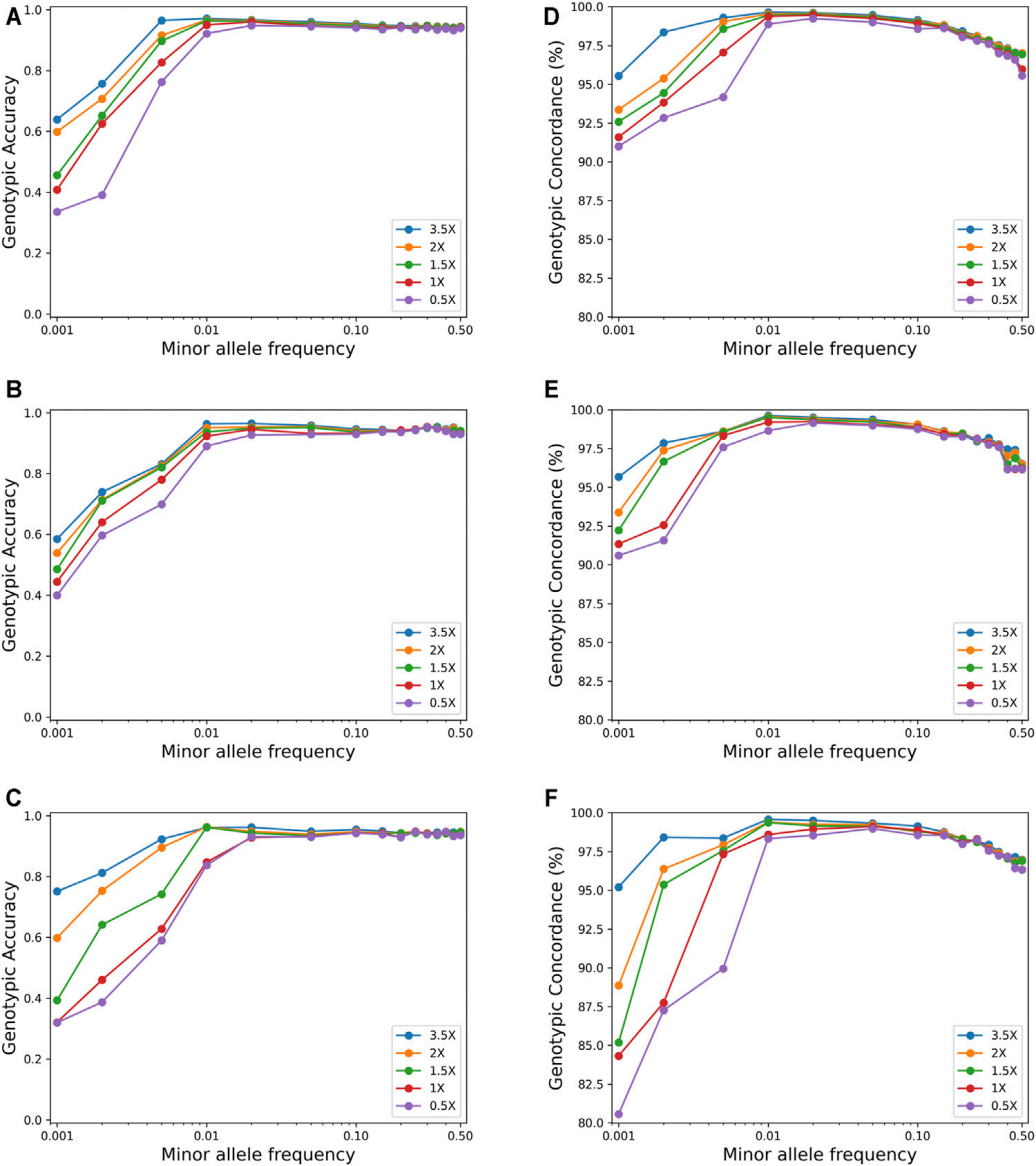
Effects of minor allele frequency on imputation genotype accuracy and genotype concordance using the pipeline of BaseVar + STITCH (sample size = 617). **(A–C)** represent genotype accuracy for chromosomes 1, 19, and 30, respectively; **(D–F)** represent genotype concordance for chromosomes 1, 19, and 30, respectively.

Figure 3 shows the effect of MAF on imputation accuracy for a sample size of 600. For SNPs with MAF <0.01, both the genotypic accuracy and the genotypic concordance were greatly affected by MAF, and the accuracy increased rapidly with the increase of MAF. However, for SNPs with MAF >0.01, the imputation accuracy was not affected by MAF, while the genotypic concordance decreased slightly with the increase of MAF.

### Variance Component Estimation

Table 1 presents the estimates of variance components and heritabilities based on the single-trait model with the two types of **G** matrix [**G**(g) and **G**(d)] constructed using five different marker sets (130, 220, 410K, and 2.3M from the 3.5x sequence data and 1.4M from the 1x sequence data). For the 3.5x sequence data, the estimates under the four marker sets were very similar, with the additive variance and heritability estimates from the 2.3M marker set being consistently slightly smaller than those from the other three marker sets. However, the additive variance and heritability estimates from the 1.4M marker set were all smaller than those from the other marker sets. For all marker sets, the estimates of additive genetic variances and heritabilities based on **G**(d) were consistently smaller than those based on **G**(g), although the differences were very small and not significant.

**TABLE 1:** Estimates of variance components and heritabilities and their standard errors (in brackets) under single-trait model using different marker sets and different **G** matrices for birth weight (BW) and weaning weight (WW).

Marker set^a^	Genotype-based G matrix			Expected dosage-based G matrix		
	*σ*_a_^2^	*σ*_e_^2^	*h* ^2^	*σ*_a_^2^	*σ*_e_^2^	*h* ^2^
BW						
130K	10.266 (2.448)	8.024 (1.730)	0.561 (0.108)	10.100 (2.409)	8.030 (1.730)	0.557 (0.108)
220K	10.424 (2.456)	7.904 (1.728)	0.569 (0.108)	10.253 (2.417)	7.912 (1.727)	0.564 (0.108)
410K	10.750 (2.457)	7.656 (1.709)	0.584 (0.106)	10.581 (2.420)	7.664 (1.709)	0.580 (0.106)
2.3M	10.166 (2.424)	8.154 (1.703)	0.555 (0.107)	10.011 (2.388)	8.159 (1.702)	0.551 (0.107)
1.4M	9.456 (2.374)	8.732 (1.692)	0.520 (0.107)	9.316 (2.340)	8.739 (1.692)	0.516 (0.107)
WW						
130K	68.419 (26.549)	139.977 (21.542)	0.328 (0.115)	68.223 (26.116)	140.874 (21.553)	0.326 (0.115)
220K	69.136 (26.566)	140.659 (20.358)	0.330 (0.115)	68.919 (26.549)	141.777 (21.542)	0.327 (0.115)
410K	69.866 (26.423)	141.046 (21.431)	0.331 (0.115)	69.670 (25.831)	141.328 (21.421)	0.330 (0.114)
2.3M	62.042 (25.269)	146.029 (20.907)	0.298 (0.112)	61.083 (24.888)	146.074 (20.900)	0.295 (0.111)
1.4M	54.305 (23.542)	152.396 (20.072)	0.263 (0.106)	53.514 (23.204)	152.422 (20.067)	0.260 (0.105)

a Marker sets 130K–2.3M were derived from the original sequence data with an average depth of 3.5x; marker set 1.4M was from sequence data with a depth of 1x.

*σ*_*a*_^*2*^, additive genetic variance; *σ*_*e*_^*2*^, residual variance; h2, heritability.

For the two-trait model, the variance and co-variance components of the two traits were estimated based on the dosage-based **G** matrix and the 410K marker set (Table 2). The estimates of heritability from the two-trait model (0.627 for BW and 0.425 for WW) were higher than those from the single-trait model (0.580 for BW and 0.330 for WW). The estimate of genetic correlation between BW and WW was 0.839.

**TABLE 2:** Estimates of variance (covariance) components, heritabilities, and genetic correlation and their standard errors (in brackets) under two-trait models using the 410K marker set and expected dosage-based G matrix.

Trait	*σ*_a_^2^	*σ*_e_^2^	*h* ^2^	Cov_a_	Cov_e_	*r*_g_	*r*_*p*_
Birth weight	11.769 (2.467)	7.016 (1.687)	0.627 (0.102)	27.399 (6.796)	9.839 (4.806)	0.839 (0.076)	0.588
Weaning weight	90.728 (25.747)	122.553 (19.855)	0.425 (0.105)				

σa2, additive genetic variance; σe2, residual variance; h2, heritability; Cova, additive genetic covariance between BW and WW; Cove, residual covariance between BW and WW; rg, genetic correlation (= $\frac{{\text{Cov}}_{\text{a}}}{\sigma_{\text{a}}\left(\text{BW}\right)\times \sigma_{\text{a}}\left(\text{WW}\right)}$ ); rp, phenotypic correlation (= $\frac{{\text{Cov}}_{\text{a}}+{\text{Cov}}_{\text{e}}}{\sqrt{\sigma_{\text{a}}^{2}\left(\text{BW}\right)+\sigma_{\text{e}}^{2}\left(\text{BW}\right)}\times \sqrt{\sigma_{\text{a}}^{2}\left(\text{WW}\right)+\sigma_{\text{e}}^{2}\left(\text{WW}\right)}}$ ).

### Accuracy and Bias of Genomic Prediction

The GEBVs for BW and WW were calculated under the single-trait model and two-trait model, respectively. For the single-trait model, we again considered both types of **G** matrix [**G**(g) and **G**(d)] constructed using the five different marker sets. The average accuracies and biases derived from 12-fold cross-validation are given in Table 3. In general, the differences in accuracy and bias between different marker sets were small and not significant, while the 410K marker set resulted in the highest accuracies, and the 1.4M marker set resulted in the lowest accuracies. No differences in prediction accuracy and bias were observed between the two types of **G** matrices. For the two-trait model, only the **G**(d) matrix constructed using the 410K marker set was used (Table 4). Compared with the results under the single-trait model with the same **G** matrix, the two-trait model remarkably improved the accuracies (0.337 vs. 0.297 for BW and 0.301 vs. 0.229 for WW) and reduced the biases (0.020 vs. 0.062 for BW and 0.117 vs. 0.169 for WW). The difference tendencies mentioned above were quite consistent across the 12 folds (see Supplementary Table S3).

**TABLE 3:** Accuracies and biases of genomic prediction and their standard errors (in brackets) under single-trait model with different marker sets.

Marker set^a^	Genotype-based G matrix		Expected dosage-based G matrix	
	Accuracy^b^	Bias^c^	Accuracy^b^	Bias^c^
Birth weight				
130K	0.285 (0.041)	0.063 (0.189)	0.285 (0.041)	0.063 (0.189)
220K	0.290 (0.040)	0.069 (0.186)	0.290 (0.040)	0.069 (0.186)
410K	0.297 (0.039)	0.061 (0.175)	0.297 (0.039)	0.062 (0.176)
2.3M	0.283 (0.039)	0.043 (0.174)	0.283 (0.039)	0.043 (0.174)
1.4M	0.277 (0.040)	0.037 (0.178)	0.277 (0.040)	0.038 (0.178)
Weaning weight				
130K	0.225 (0.031)	0.163 (0.165)	0.225 (0.031)	0.164 (0.166)
220K	0.226 (0.032)	0.163 (0.166)	0.226 (0.031)	0.163 (0.165)
410K	0.229 (0.031)	0.168 (0.163)	0.229 (0.031)	0.169 (0.163)
2.3M	0.223 (0.032)	0.149 (0.179)	0.223 (0.032)	0.149 (0.179)
1.4M	0.221 (0.031)	0.183 (0.178)	0.221 (0.031)	0.183 (0.178)

a Marker sets 130K–2.3M were derived from the original sequence data with an average depth of 3.5x; marker set 1.4M was from sequence data with a depth of 1x.

b Accuracy is defined as the correlation between GEBVs and corrected phenotypes (*y*_c_).

c Bias is defined as 1-regression coefficient of GEBVs on *y*_c_.

**TABLE 4:** Accuracies and biases of genomic prediction and their standard errors (in brackets) under single-trait and two-trait models.

Model^a^	Birth weight		Weaning weight	
	Accuracy^b^	Bias^c^	Accuracy^b^	Bias^c^
Two-trait	0.337 (0.037)	0.020 (0.141)	0.301 (0.038)	0.117 (0.164)
Single-trait	0.297 (0.039)	0.062 (0.176)	0.229 (0.031)	0.169 (0.163)

a For the two-trait model, only the expected dosage-based **G** matrix constructed using the 410K marker set derived from the 3.5x sequence data was used. For comparison, the results of the single-trait model using the same **G** matrix is represented here.

b Accuracy is defined as the correlation between GEBVs and corrected phenotypes (*y*_c_).

c Bias is defined as 1-regression coefficient of GEBV on *y*_c_.

## Discussion

Low-coverage whole genome sequencing followed by imputation provides a cost-effective way for genome-wide high-density genotyping, especially for species (such as donkey) for which a SNP array is not available. In this study, we investigated the strategies for genotype imputation and evaluated the performance of genomic prediction using imputation-based sequence data in a donkey population.

### Strategies of Imputation for Low-Coverage Sequence Data

Imputation is necessary for lcWGS data due to the high missing rates, which involves two steps, i.e., SNP calling and imputation. A proper pipeline is essential to ensure high imputation performance. In this study, we compared two pipelines, Bcftools + Beagle and BaseVar + STITCH. In the first pipeline, both Bcftools and Beagle have been widely used for SNP calling and imputation for sequence data, respectively. However, it is not clear whether they are suitable for lcWGS data. On the other hand, BaseVar and STITCH were designed specifically for lcWGS data. We demonstrated that BaseVar + STITCH outperformed Bcftools + Beagle (Figure 1). Furthermore, we showed that in our Dezhou donkey population, using this pipeline, high imputation accuracy (genotypic accuracy >0.94 and genotypic concordance >98%) can be achieved with a sample size of 400 and a sequencing depth of 1x (Figure 2). Similar results were also reported by Zhang et al. (2021). In other words, with a sample size of over 400, a sequencing depth of 1x could be sufficient to ensure high imputation accuracy using BaseVar + STITCH.

### Genomic Prediction Using Imputation-Based Sequence Data

Using the imputation-based sequence data, we evaluated the performance of genomic prediction using GBLUP with respect to two types of **G** matrices [**G**(g) and **G**(d)], five different marker sets (130, 220, 410K, and 2.3M derived from the 3.5x sequence data and 1.4M derived from the 1x sequence data), and single-vs. two-trait GBLUP model.

#### Comparison of the Two Types of **G** Matrices

We found that the accuracies and biases of genomic prediction derived from the two types of **G** matrices were almost the same in all scenarios. Note that the variance component estimates from the two types of **G** matrices were also very similar. This implicates that for our given data, the two types of **G** matrices did not lead to different results. It remains to be seen whether this results also holds for other data sets.

#### Comparison of the Five Marker Sets

For the four marker sets from the 3.5x sequence data, the prediction accuracy increased slightly (although not significant) when the marker density increased from 130 to 410K, but did not further increase when the density increased to 2.3M. The densities of 130, 220, and 410K correspond to medium to high density of SNP array, while the 2.3M corresponds to the density of sequence data. Some studies showed that, in the frame of GBLUP, the genomic prediction accuracy could be improved using high-density SNP array compared to using medium-density array (VanRaden et al., 2011; Su et al., 2012; Perez-Enciso et al., 2015), but there were also studies that showed no or very small such improvement (VanRaden et al., 2013; Boison et al., 2017). It has been shown that, in the frame of GBLUP, using sequence data could hardly improve the accuracy compared with using SNP array (Ober et al., 2012; Perez-Enciso, 2014; van Binsbergen et al., 2015; Frischknecht et al., 2018). However, this does not mean that sequence data is of no value for genomic prediction. Several studies have shown that sequence data would be beneficial when variants are preselected based on, e.g., GWAS or a Bayesian selection model (MacLeod et al., 2016; Hayes and Daetwyler, 2019). In addition, sequence data can be meaningful for cross-breed/population genomic selection (Druet et al., 2014; MacLeod et al., 2016). On the other hand, the prediction accuracies using the 1.4M marker set from the 1x sequence data were slightly lower than those from the 3.5x sequence data. This should be due to the lower imputation accuracy for the 1x sequence data than 3.5x (see Figure 2). However, since the reduction in accuracy was rather small, in consideration of the sequencing cost, sequencing at depth of 1x would be a preferred choice for a lcWGS-based genomic selection.

#### Single-vs. Two-Trait GBLUP Model

Noticeable increases in genomic prediction accuracy were observed when using a two-trait model compared with using a single-trait model. The comparison was made only for the scenario of using an expected dosage-based **G** matrix and the 410k marker set derived from the 3.5x sequence data. However, such advantage should hold for other scenarios. It has been shown in several incidences that a multi-trait model can increase the accuracy of breeding value estimation, either by conventional BLUP or by GBLUP (Calus and Veerkamp, 2011; Jia and Jannink, 2012; Guo et al., 2014), in particular for traits with high genetic correlation, such as the two traits investigated in this study. This increase in accuracy with multi-trait model will be particularly beneficial for the situation where the reference population size is limited.

It should be pointed out that, although the differences in the performance of genomic prediction between different scenarios seemed reasonable, some of the differences were actually not significant, possibly due to the small dataset available for this study. It is the practical situation for some species/breeds/populations for which only a small dataset is available for investigating genomic prediction. Therefore, despite the limitations of having a small dataset, our findings would provide meaningful inspirations for such situations.

## Conclusion

In this study, we demonstrated that the pipeline BaseVar + STITCH is a good choice for SNP calling and imputation for low-coverage sequence data. A sufficient high imputation accuracy could be achieved for sequence data with a sequencing depth as low as 1x, when the size of the sequencing population is over 400. Thus, lcWGS combined with imputation provides a cost-effective way for whole genome high-density genotyping and can be applied for large-scale genomic selection in farm animals. This is particularly beneficial for those animal species for which a SNP array is not available. In the frame of GBLUP, increasing marker density from a density comparable with a high-density SNP array (e.g., 400K) to sequence density with millions of SNPs did not increase the accuracy of genomic prediction. The multi-trait model GBLUP improves the accuracy of genomic prediction over the single-trait model, which would be particularly meaningful for the situation where the reference population size is limited.

## Data Availability

The datasets presented in this study can be found in online repositories. The names of the repository/repositories and accession number(s) can be found in the article/Supplementary Material.

## Supplementary Material

Supplementary Figure S1 The number of individuals of different sequencing depths. The sequencing was performed in two batches. The first batch consisted of 317 animals and the average sequencing depth was 4.5x; the second batch consisted of 300 animals and the average depth was 2.4x. Taken together, the overall average depth was 3.5x.
