# Supplementary figures and images for: Towards a Cost-Effective Implementation of Genomic Prediction Based on Low Coverage Whole Genome Sequencing in Dezhou Donkey
Source: Front Genet. 2021 Nov 3;12:728764. doi: 10.3389/fgene.2021.728764 (PMC8595392; doi:10.3389/fgene.2021.728764)

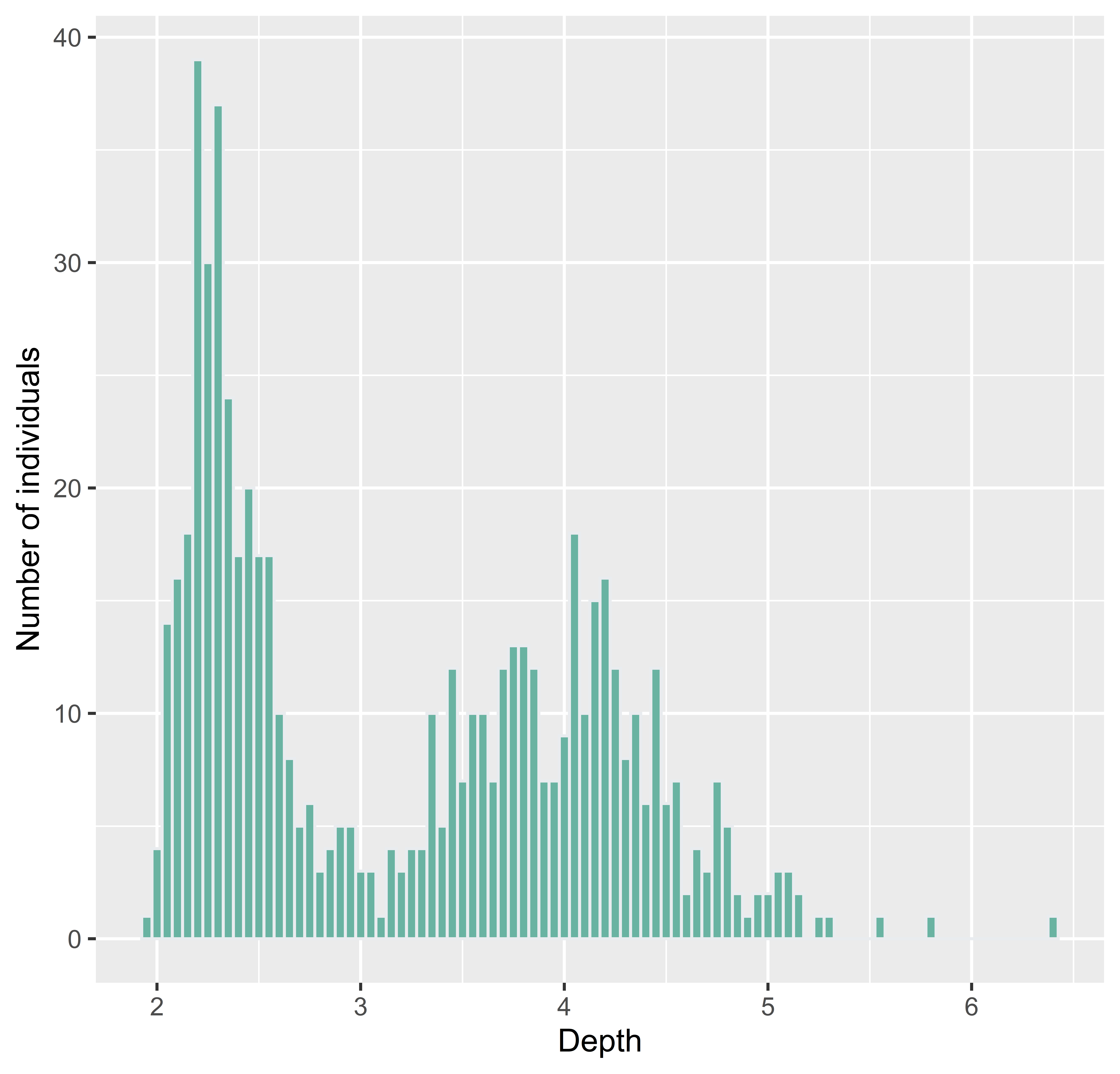

Supplement: Supplementary file 3 [file Image1.TIF]
